# The physician's unique role in preventing violence: a neglected opportunity?

**DOI:** 10.1186/1741-7015-10-146

**Published:** 2012-11-23

**Authors:** John C Umhau, Karysse Trandem, Mohsin Shah, David T George

**Affiliations:** 1 Laboratory of Clinical and Translational Studies, National Institutes of Health, National Institute on Alcohol Abuse and Alcoholism, 10 Center Drive, Building 10-CRC Hatfield Center, Room 1-5330, Bethesda, MD, 20892-1108, USA; 2 Department of Obstetrics and Gynecology, Grand Itasca Clinic and Hospital, 1601 Golf Course Road, Grand Rapids, MN 55744, USA; 3 Medical College, CMH Lahore Medical College, University of Health Sciences, Abdur Rahman Road, Cantt, Lahore, Punjab, 54000, Pakistan

**Keywords:** aggression, alcoholism, psychopharmacology, violence, domestic abuse, prevention, neurobiology, treatment, domestic violence

## Abstract

**Background:**

Episodes of explosive rage and violence comprise a symptom complex which can have a devastating effect on a person's life. In the community this behavior is seen as workplace violence, domestic abuse and road rage, while in the clinical setting, this behavior is rarely mentioned by patients, despite evidence that it can signify an important biological disorder that may afflict more than three percent of the population.

**Discussion:**

Patients are often reluctant to seek help for episodic attacks of rage, especially attacks which are accompanied by physical violence. Although, in the past, clinicians have had few treatment options to offer, recent neuroscience advances have created new possibilities to understand and help patients with this neglected problem. No formal medical guidelines for treating violence exist; however, many patients can be helped by diagnosis, referral and treatment. Treatment can include pharmaceuticals and nutrients, as well as referral for anger management or behavioral therapy.

**Summary:**

The astute clinician has an opportunity to positively impact an important problem through the diagnosis and treatment of patients with symptoms of intermittent explosive disorder.

## Background

The symptom complex characterized by repeated episodes of explosive rage and violence, disproportionate to any provocation, is rarely a patient's chief complaint, but it is a common problem none the less. For the afflicted individual, such a complaint may have profound effects on their self-esteem, their interpersonal relationships, and even on their ability to function in society. While some violence can be premeditated, goal directed or related to antisocial, callous unemotional traits, it is rather the category of violence described as affective aggression, (that is, reactive, defensive or hostile aggression) associated with fear and threat which will be the focus of this paper [1]. For most clinicians, the clinical perspective of violence as seen with Intermittent Explosive Disorder, specified by DSM-IV to encompass "several discrete episodes of failure to resist aggressive impulses that result in serious assaultive acts or destruction of property", with the further specification that "the degree of aggressiveness expressed during the episodes is grossly out of proportion to any precipitating psychological stressors" [2], provides a framework for addressing this issue from a clinical standpoint. When afflicted individuals express a concern about this unwanted behavior, it becomes an issue which clearly warrants the concern of their physician. This paper will give a brief review of the clinical approach to this, often ignored, yet common problem.

Intermittent explosive disorder has a prevalence of 3.9%, suggesting that each year in the United States, it is responsible for more than 30 million violent assaults [3]. When manifested as domestic abuse, road rage or workplace violence, such behavior is commonly attributed to cultural factors, social stress, economic deprivation or substance abuse. Much more needs to be known about these behaviors; however, recent advances in neuroscience allow for a more complete understanding, which accounts for biological factors amenable to medical treatment [4-8].

A number of factors have limited the benefits which a neurobiological perspective on violence might bring. Perpetrators may be unaware that they could benefit from medical intervention. Clinicians may be uncomfortable dealing with an area of diagnosis and treatment with which they are unfamiliar. Considering a violent perpetrator to be responsible for their actions may be a critical legal policy to uphold, but such a policy may impede diagnosis. All these factors ultimately work together to deny the benefits of modern clinical care to individuals who may urgently need it.

This article will provide a brief overview of the diagnosis and treatment of chronic violent behavior. A brief discussion of the neurobiology of violence will be presented and legal aspects of treatment will also be discussed in an effort to encourage clinical interest in what may be one of the most neglected areas of medicine.

## Discussion

### Neurobiology

One way to understand the biological basis for explosive types of rage and violence is to consider it as behavior which promotes survival in the face of threat [6]. Although many "threats" are psychological, rather than physical, the autonomic nervous system is aroused in either case. The resulting rage response is an innate behavior that rises from the limbic system (for example, amygdale and related structures) and is associated with palpitations, sweating, tachypnea and trembling [9,10]. The individual's own perception of these symptoms may incite further arousal in a positive feedback loop on the limbic system response. The prefrontal cortex and the anterior cingulate gyus provide a "top-down" inhibition of this limbic response while evaluating whether a physical or psychological "threat" is a harmless insult or a serious challenge to survival [9]. Controlling this limbic response appropriately is one of the most time critical functions of the frontal cortex; the serotoninergic neurotransmitter system is thought to be critical in this process [11]. The rapid appraisal of potential threats is especially sensitive to perturbation, such as the sensory processing distortions caused by metabolic disturbances or substance abuse [9]. It is the prefrontal cortex which allows for the consideration of nuanced and non-violent responses to any specific "threat" [12]. However, when violence is called for, limbic (for example, amygdale) activation will shut down the calming input from the prefrontal cortex in order to promote quick reactions and efficient fighting [13].

Various neurological disorders can disrupt the critical and sensitive processing necessary for appropriate behavior in response to perceived threat [9]. Neuroimaging studies suggest that in the pathological situation, calming cortical inputs are shut down when they should not be, permitting exaggerated and inappropriate limbic arousal and rage [8,12]. Impaired control of the limbic system rage response in this situation explains how a domestic violence perpetrator could physically attack their spouse with no apparent concern for the consequences. If cortical control is dysfunctional, relatively minor slights to the ego might be misinterpreted as overwhelming threat, leading to a violent response [12]. Acute psychological stress as well as social and cultural factors may interact with neurobiological deficits to create a situation where inappropriate violence becomes frequent [14].

### Diagnosis

Episodes of explosive rage and violence can be a defining feature of a patient's personal life; however, in the same way that patients can be reluctant to admit they have depression; they are even more ashamed to admit they have violent behavior. Often patients experience remorse, but not always. Simply asking during a history and physical examination, "Do you lose your temper? Do you do things when you are angry that you later regret?" can lead an individual to discuss behaviors that they would otherwise never initiate. If a clinician can suspend judgment about their patient's behavior and discuss situations without making disparaging remarks, patients can potentially overcome their denial and face the destructive effects of their "anger problem". The use of valid scales, such as the STAXI [15] and the BPAQ-SF [16] may also be helpful in identifying such patients. Between episodes, there is no classical way for someone with rage and violence to present for care; however, considering the association between violence and alcohol, individuals undergoing alcoholism detoxification may be more likely to benefit from screening. Impulsive aggression is frequently found in a co-morbid association with substance abuse and with personality disorders. Also, patients presenting with injuries suffered as a result of interpersonal violence are themselves at risk for having inappropriate attacks of rage [17]. When patients do present with violence, it is a common cause for involuntary commitment to a psychiatric facility and acute therapy [18,19].

Although in many cases violence may be a behavior simply learned from the patient's social and cultural background, the unique role of the clinician is to critically evaluate each patient for biological factors which can be treated, or refer the patient to a physician who can [20-24]. Many disorders which affect the central nervous system have been associated with violence [20,25-29]. Episodic attacks of rage and violence are not a classic presentation of any specific disorder, but this behavior can arise from diverse causes, including epilepsy, nutritional deficiencies, brain tumors, toxic exposures and encephalopathy from endocrine, metabolic or infectious disorders [18]. Closed head injuries can leave patients with no objective sign of their injury but with a dramatic predilection for episodes of impulsive rage [27]. Both acute and chronic substance abuse, for example, alcohol, and stimulants, can contribute to violence [30]. Depression, mania, post-traumatic stress disorder and schizophrenia may be associated with irritably, rage and violence [20]. (See Table 1)

**Table 1:** Some medical conditions associated with episodic rage and violence

Psychiatric	Organic brain syndrome [35], Korsakoff's psychosis [86], bipolar disorder [87], psychoactive substance intoxication and withdrawal [88], psychotic disorders [89], premenstrual dysphoric disorder [90], post-traumatic stress disorder [22], panic disorder [4], generalized anxiety disorder [91], antisocial personality disorder [92], borderline personality disorder [92], personality disorders [92], attention deficit disorder [93], major depressive disorder [94].
**Neurological**	Brain tumors/lesions [95], head injury [27], Parkinson's disorder [96], seizure disorder [26], multiple sclerosis [97], normal pressure hydrocephalus [98], autism [34], Wilson's disease [29], Huntington's disease [99], dementia [100], stroke [101].

**Nutritional deficiency**	Omega-3 fatty acids [56], folate [29], cobalamin (B_12_) [102,103], thiamine [104,105], iron [106], lithium [107], magnesium [53,108], tryptophan [109], pyridoxine [29].

**Medical and toxic**	Alcohol [21,110], cocaine [21,111], manganese [29,112], organophosphorous compounds [29,113], phencyclidine (PCP) [114], lysergic acid diethylamide (LSD) [101,115], heroin [101,116], amphetamines [117], benzodiazepines [117,118], prescription drugs [117,119], anabolic steroids [117].

**Metabolic/Endocrine**	Hypoglycaemia [101,117,120], hepatic encephalopathy [121], fluid and electrolyte imbalances [119], disorders of the thyroid, parathyroid and adrenal glands [29,122], hypoxia [101].

**Miscellaneous**	Intermittent Porphoria [123], chronic pain [124], sleep disorders [29,125], anemia [126], vasculitis [119], meningitis/encephalitis [101], syphilis [102].

It is important to note that not all instances of violence have serious underlying pathology. For example, the aggression associated with hypoglycemia can be simply treated with food. Identifying the precise biological factors contributing to violence can be difficult as multiple predisposing conditions often coexist in one patient. As with any neurological issue, diagnostic testing is led by the symptom presentation and physical exam. For example, if temporal lobe seizures are suspected, an electroencephalogram (EEG) may be indicated [26]. In most cases, however, the physical exam will be normal, and a precise diagnosis will be elusive. In such a situation, empiric treatment may be indicated.

### Treatment

Although no drugs have a Food and Drug Administration (FDA) approved indication for violence, many therapeutic options have been tested in randomized, placebo-controlled double-blind trials [20,28,31]. (see Table 2) Psychiatrists use various classes of drugs as treatment for violence, but treatment guidelines have not been established. The point of this article is to introduce existent therapeutic options rather than to provide a detailed review of pharmacotherapy, which has been reviewed elsewhere [18,20,25,31-40]. Medication is not a panacea; all types of violence are not amenable to pharmacological therapy. For example, treatment with the classic anticonvulsant phenytoin was shown to reduce impulsive but not premeditated violence [41]. In previous reviews the use of anticonvulsants, beta blockers and atypical antipsychotic agents has been described in detail [20,31,40-49]. For more than 40 years, lithium has been used in many populations to treat violence [50]. Serotonin reuptake inhibitors are commonly used in the setting of depression but can also be effective for reducing chronic anger [20]. Serotonin reuptake inhibitors are often initially used at low doses to guard against any paradoxical exacerbating effect [31,22,41]. Since the use of all of these medications for reducing violence is "off label", it is important to carefully monitor for any adverse effects which can in some instances cause an increase in irritability and aggression [22,40].

**Table 2:** Examples of some agents used to reduce violence and aggression

Medications for alcoholism	Naltrexone and acamprosate in sober alcoholics to reduce craving and relapse [63,64]; prazosin in alcoholism with PTSD for caving and relapse [127].
**Nutritional factors**	Essential omega 3 fatty acids, docosahexaenoic acid (DHA) and eicosapentaenoic acids (EPA) in bipolar disorder and borderline personality disorder; multiple vitamins and minerals with DHA and EPA in young male prisoners, and in children [53,57-59,128].

**Lithium**	In children and adults, in personality disorders, and in combination with other anticonvulsants or atypical antipsychotics [40,48].

**Beta-blockers**	Propranolol in brain-injury, autism, schizophrenia, organic mental disorders [37,129].

**Anticonvulsants**	Carbamazepine/oxcarbazepine in impulsive violence and in elderly [32,36]; phenytoin in prisoners and in intermittent explosive disorder [32,38,49,130]; Topriamate in borderline personality disorder and in depression [40]; valproate/divalproex in a variety of psychiatric conditions [32,40].

**Antidepressants**	Flouxetine in personality disorders [131], and in domestic abuse when combined with Cognitive Behavioral Therapy (CBT) and alcohol treatment [132]; trazodone in dementia associated aggression [133].

**Atypical antipsychotics**	Clozapine in schizophrenia [33,134]; quetiapine in schizophrenia and bipolar disorder [33,40]; Loxapine in schizophrenic and psychosis [40], olanzapine in schizophrenia and borderline personality disorder [33,40]; aripiprazole in schizophrenia, autism spectrum disorders and borderline personality disorder [33,40]; risperidone in a variety of psychiatric illness [40].

Nutritional factors, especially the essential omega-3 fatty acids, docosahexaenoic acid (DHA) and eicosapentaenoic acid (EPA) are critical components of the nervous system, but can be deficient in the diet [51-53]. Some controlled trials suggest that these nutrients may reduce aggression and produce other benefits with few side effects [54-56]. In recognition of this, the American Psychiatric Association recommends that individuals with mood, impulse-control or psychotic disorders consume 1 gram of EPA + DHA per day [57]. When essential fatty acids were administered with multiple vitamins to young men in prison, violence was reduced by one third [58], a finding which was later replicated [59]. Although there are many more studies demonstrating the effectiveness of prescription pharmaceuticals than there are of studies testing nutrients, omega-3 fatty acid (that is, fish oil) and vitamins have the benefit that they may be readily accepted by patients who desire a more "natural" type of therapy without the stigma and serious side effects which sometimes accompany psychotropic medications [60]. Because of the potential for nutritional therapy to address the problem of violence on a public health basis, there is a critical need for more research to resolve controversies in this area.

### Substance abuse treatment as therapy for aggressive behavior

Alcoholism may be the most common condition associated with violence; alcohol is thought to have a disinhibiting effect on aggressive behavior [6]. Reports suggest that most severe interpersonal violence occurs when the perpetrator is intoxicated. Cocaine abuse is also associated with violence [30]. Not surprisingly, recent research has found that substance abuse treatment can reduce the prevalence of violent behavior by more than half [61]. Patients can be referred to specific substance abuse treatment programs as well as to self-help programs such as Alcoholics Anonymous. Medical management of alcoholism is effective and can include the use of naltrexone or acamprosate [62]. These drugs are relatively underused medications which can reduce the incidence of relapse to alcoholic drinking [63]. Side effects are relatively minor, and in some alcoholics, these drugs can have a marked effect [64].

### Psychological intervention as a treatment for aggression

In addition to any prescribed medical treatment, patients must recognize their need to change, assume responsibility for their behavior and begin to deal with conflict in a positive manner. To facilitate these cognitive processes, violent individuals are commonly offered anger management or behavioral therapies [65-68].

Psychological interventions can be very helpful for some aggressive individuals [69], and effects can last for months, even years after the therapy is terminated [66-68]. In a meta-analysis looking at 50 studies, angry, (and in some cases violent), patients treated with a variety of cognitive behavioral therapy (CBT) treatments had better outcomes than 76% of non-treated control patients [70]. These studies considered inpatients and outpatients, children and adults, abusive parents and spouses, prisoners and individuals with mental retardation as well as the general population. The most effective CBT anger management programs include two or all three of the following components: relaxation techniques, cognitive interventions and communication skills [71]. For individuals with borderline personality disorder, dialectical-behavioral therapy (DBT) is an effective therapy [72,73]. The success of psychological interventions on domestic violence recidivism is controversial, but a meta-analysis of 22 studies suggests that the effect size of therapy is generally significant though small [65]. Treatments specifically for domestic violence include feminist psycho-educational men's groups, (that is, the Duluth model) [68], men's CBT groups, anger management and couples' therapy [65]. Many states regulate the therapy of domestic abusers and, in some cases, restrict the use of couple's therapy or the consideration of neuro-psychiatric issues (for example, alcoholism); the extent to which such legislation inhibits potentially beneficial treatment has not been evaluated [74]. Recently, a computer assisted treatment adjunct based on a Trans Theoretical Model of behavior change has been shown to reduce violence, suggesting that entirely new treatment paradigms could be developed in the future [75]. The effectiveness of domestic violence treatment is difficult to study, and the problem is compounded by the effect of high dropout rates (40% to 60% within three months), which predict poor outcome [69]. A positive correlation has been found repeatedly between the strength of the therapeutic alliance, patient attendance rates and the overall likelihood of the participant benefiting from the treatment [76,77]. Therefore, when referring aggressive patients for therapy, a physician should supply several options and emphasize the importance of finding a therapist with whom the patient feels comfortable. In the United States, the American Psychological Association (1-800-964-2000) or the National Domestic Violence Hotline (1-800-799-SAFE) can provide referral information.

### Legal and ethical issues in the United States

While the American Medical Association's Ethics Code requires and emphasizes the importance of patient confidentiality, it also states the following: "When a patient threatens to inflict serious bodily harm to another person or to him or herself and there is a reasonable probability that the patient may carry out the threat, the physician should take reasonable precautions for the protection of the intended victim, which may include notification of law enforcement authorities..." (E-5.05 Confidentiality). Additionally, in the landmark Tarasoff cases (Tarasoff v. Regents of the University of California 1974 and 1976) [78] the ruling established in the state of California a legal duty of health workers to warn and protect potential victims from a patient's foreseeable violence. Since then, some states have codified the health worker's duty to protect specifying how to discharge that duty, some states have declined to uphold the duty to protect in court and some states have not addressed the issue in court or legislation [79]. In states where the duty to protect does apply it is only operative when the patient identifies a specific victim or has a history of violence and there is substantial and compelling reason to believe that the patient will be violent again. The health worker fulfils the duty in most states by informing the relevant authorities, warning the victim or having the patient hospitalized, although a few states have additional requirements. For victims who are minors, all 50 states have passed some form of a mandatory abuse and neglect reporting law. Some states mandate that the suspicion of domestic violence or elder abuse must be reported to the legal authorities [80]. All states require reporting of suspected child abuse [81]. When treating an adult victim, mandatory reporting laws also vary by state and are based on the type and circumstances of the injury; laws in all states are listed in a 2002 article by Houry and colleagues [82]. For more information on legal responsibilities as a physician, the American Medical Association recommends contacting the state licensing board. Screening a patient for violent activity can be unethical if a physician fails to discuss the limits of confidentiality and any legal requirement of the state to break confidentiality and involve the legal system [83].

While knowledge of laws in one's state of practice is essential, experts in medical law strongly recommend that clinicians approach the dangerousness of a patient as a clinical rather than legal issue [79,84,85]. The primary determinants in a malpractice suit are the thoroughness of the clinicians' assessment of a patient's potential for violence and the care with which options of action were considered, rather than the accuracy of the clinicians' predictions [79]. Thorough mental status examinations and substance use histories should be documented with direct quotes from the patient. Careful notes should be taken explaining why a certain course of action was taken, what other options were considered but opted against and why. The more potentially dangerous the client, the more care one should take in the documentation of the case [85].

## Summary

Although it is unknown what proportion of explosive rage and violent behavior can be successfully treated, the price of ignoring treatment includes the staggering cost of injuries that might be prevented, and an even greater cost in shattered communities, broken families and personal suffering. Various neurobiological disorders can affect the processing of perceived threats by the brain, leading to dysfunctional, violent behaviors. Future research will add to our understanding of violence, but existing evidence clearly shows that physicians today have a unique opportunity to address medical aspects of this problem when they identify the perpetrators of explosive violent behavior. Asking patients if they lose their temper can identify violent individuals in a non-judgmental way and can open the door to appropriate medical evaluation, treatment and referral. Patients are given an important degree of self-determination when they are offered expanded treatment options that include not only behavioral therapy, but also nutritional supplementation, alcoholism treatment and pharmaceuticals.

## Abbreviations

CBT: Cognitive Behavioral Therapy; DBT: dialectical behavior therapy; DHA: docosahexaenoic acid; EPA: eicosapentaenoic acids; FDA: Food and Drug Administration.

## Competing interests

The authors declare that they have no competing interests.

## Authors' contributions

JCU conceptualized, wrote and edited the manuscript. KT and MS wrote and edited the manuscript. DTG provided intellectual background and assisted in writing and editing the manuscript. All authors read and approved the final manuscript.

## Authors' information

The authors alone are responsible for the content and writing of this commentary. JCU is certified in both Public Health and Addiction Medicine, and conducts clinical research in neuroscience at the National Institutes of Health where he has studied violence, nutrition, and alcoholism. He has more than 20 years' experience treating homeless patients. KT is certified in Obstetrics and Gynecology. MS is a medical student from CMH Lahore Medical College, University of Health Sciences, Lahore, Pakistan. DTG is certified in Psychiatry and conducts clinical research at the National Institutes of Health where he has studied domestic violence and new treatments for alcoholism.

## Pre-publication history

The pre-publication history for this paper can be accessed here:

http://www.biomedcentral.com/1741-7015/10/146/prepub

## References

[B1] Siegel A Victoroff J Understanding human aggression: new insights from neuroscience Int J Law Psychiatry 2009 32 209 215 10.1016/j.ijlp.2009.06.001 19596153

[B2] American Psychiatric Association Task Force on D-I Diagnostic and statistical manual of mental disorders: DSM-IV-TR 2000 Arlington, VA: American Psychiatric Publishing, Inc.

[B3] Kessler RC Coccaro EF Fava M Jaeger S Jin R Walters E The prevalence and correlates of DSM-IV intermittent explosive disorder in the National Comorbidity Survey Replication Arch Gen Psychiatry 2006 63 669 678 10.1001/archpsyc.63.6.669 16754840 PMC1924721

[B4] George DT Anderson P Nutt D Linnoila M Aggressive thoughts and behavior: another symptom of panic disorder? Acta Psychiatr Scand 1989 79 500 502 10.1111/j.1600-0447.1989.tb10294.x 2750551

[B5] George DT Hibbeln JR Ragan PW Umhau JC Phillips MJ Doty L Hommer D Rawlings RR Lactate-induced rage and panic in a select group of subjects who perpetrate acts of domestic violence Biol Psychiatry 2000 47 804 812 10.1016/S0006-3223(99)00300-5 10812039

[B6] George DT Phillips MJ Doty L Umhau JC Rawlings RR A model linking biology, behavior and psychiatric diagnoses in perpetrators of domestic violence Med Hypotheses 2006 67 345 353 10.1016/j.mehy.2006.01.049 16580153

[B7] George DT Umhau JC Phillips MJ Emmela D Ragan PW Shoaf SE Rawlings RR Serotonin, testosterone and alcohol in the etiology of domestic violence Psychiatry Res 2001 104 27 37 10.1016/S0165-1781(01)00292-X 11600187

[B8] George DT Rawlings RR Williams WA Phillips MJ Fong G Kerich M Momenan R Umhau JC Hommer D A select group of perpetrators of domestic violence: evidence of decreased metabolism in the right hypothalamus and reduced relationships between cortical/subcortical brain structures in position emission tomography Psychiatry Res 2004 130 11 25 10.1016/S0925-4927(03)00105-7 14972365

[B9] Siever LJ Neurobiology of aggression and violence Am J Psychiatry 2008 165 429 442 10.1176/appi.ajp.2008.07111774 18346997 PMC4176893

[B10] Davis M Rainnie D Cassell M Neurotransmission in the rat amygdala related to fear and anxiety Trends Neurosci 1994 17 208 214 10.1016/0166-2236(94)90106-6 7520203

[B11] Davidson RJ Putnam KM Larson CL Dysfunction in the neural circuitry of emotion regulation--a possible prelude to violence Science 2000 289 591 594 10.1126/science.289.5479.591 10915615

[B12] Bufkin JL Luttrell VR Neuroimaging studies of aggressive and violent behavior: current findings and implications for criminology and criminal justice Trauma Violence Abus 2005 6 176 191 10.1177/1524838005275089 15753199

[B13] Garcia R Vouimba RM Baudry M Thompson RF The amygdala modulates prefrontal cortex activity relative to conditioned fear Nature 1999 402 294 296 10.1038/46286 10580500

[B14] Eysenck HJ The origins of violence J Med Ethics 1979 5 105 107 10.1136/jme.5.3.105 490568 PMC1154734

[B15] Forgays DG Forgays DK Spielberger CD Factor structure of the State-Trait Anger Expression Inventory J Pers Assess 1997 69 497 507 10.1207/s15327752jpa6903_5 9501480

[B16] Diamond PM Magaletta PR The short-form Buss-Perry Aggression Questionnaire (BPAQ-SF): a validation study with federal offenders Assessment 2006 13 227 240 10.1177/1073191106287666 16880276

[B17] Dutton DG My back pages: reflections on thirty years of domestic violence research Trauma Violence Abus 2008 9 131 143 10.1177/1524838008319146 18541698

[B18] Simon RI Tardiff K Textbook of Violence Assessment and Management 2008 355 Arlington, VA: American Psychiatric Publishing

[B19] Rossi J Swan MC Isaacs ED The violent or agitated patient Emerg Med Clin North Am 2010 28 235 256 x 10.1016/j.emc.2009.10.006 19945609

[B20] Fava M Psychopharmacologic treatment of pathologic aggression Psychiatric Clin North Am 1997 20 427 451 10.1016/S0193-953X(05)70321-X 9196923

[B21] Lane SD Kjome KL Moeller FG Neuropsychiatry of aggression Neurologic Clin 2011 29 49 64 vii 10.1016/j.ncl.2010.10.006 PMC3053027 21172570

[B22] Mattson M editor Neurobiology of aggression: understanding and preventing violence 2003 Totowa, NJ: Humana Press

[B23] Olvera Rene L Intermittent explosive disorder: epidemiology, diagnosis and management CNS drugs 2002 16 517 526 10.2165/00023210-200216080-00002 12096933

[B24] Potter-Efron RT Differential diagnosis of physiological, psychiatric and sociocultural conditions associated with aggression and substance abuse J Chem Dependency Treat 1990 3 37 59 10.1300/J034v03n01_02

[B25] Schneider LS Sobin PB Non-neuroleptic medications in the management of agitation in Alzheimer's disease and other dementia: a selective review Int J Geriatr Psychiatry 1991 6 691 708 10.1002/gps.930061003

[B26] Lewis DO Pincus JH Epilepsy and violence: evidence for a neuropsychotic-aggressive syndrome J Neuropsychiatry Clin N 1989 1 413 418 10.1176/jnp.1.4.413 2521094

[B27] Rosenbaum A Hoge SK Head injury and marital aggression Am J Psychiat 1989 146 1048 1051 2750978 10.1176/ajp.146.8.1048

[B28] Tardiff K Concise Guide to Assessment and Management of Violent Patients 1996 Arlington, VA: American Psychiatric Publishing, Inc.

[B29] Tardiff K Unusual diagnoses among violent patients Psychiatr Clin North Am 1998 21 567 576 10.1016/S0193-953X(05)70023-X 9774796

[B30] Chermack ST Blow FC Violence among individuals in substance abuse treatment: the role of alcohol and cocaine consumption Drug Alcohol Depend 2002 66 29 37 10.1016/S0376-8716(01)00180-6 11850133

[B31] Goedhard LE Stolker JJ Heerdink ER Nijman HL Olivier B Egberts TC Pharmacotherapy for the treatment of aggressive behavior in general adult psychiatry: a systematic review J Clin Psychiatry 2006 67 1013 1024 10.4088/JCP.v67n0702 16889443

[B32] Huband N Ferriter M Nathan R Jones H Antiepileptics for aggression and associated impulsivity Cochrane Database Syst Rev 2010 CD003499 20166067 10.1002/14651858.CD003499.pub3 PMC4163499

[B33] Bitter I Czobor P Dossenbach M Volavka J Effectiveness of clozapine, olanzapine, quetiapine, risperidone, and haloperidol monotherapy in reducing hostile and aggressive behavior in outpatients treated for schizophrenia: a prospective naturalistic study (IC-SOHO) Eur Psychiatry 2005 20 403 408 10.1016/j.eurpsy.2005.01.009 16084068

[B34] McDougle CJ Stigler KA Posey DJ Treatment of aggression in children and adolescents with autism and conduct disorder J Clin Psychiatry 2003 64 16 12672261

[B35] Fleminger S Greenwood R Oliver D Pharmacological management for agitation and aggression in people with acquired brain injury Cochrane Database Syst Rev 2006 CD003299 17054165 10.1002/14651858.CD003299.pub2 PMC13735122

[B36] Stanford M Anderson N Lake S Baldridge R Pharmacologic treatment of impulsive aggression with antiepileptic drugs Curr Treat Options Neurol 2009 11 383 390 10.1007/s11940-009-0043-3 19744405

[B37] Haspel T Beta-blockers and the treatment of aggression Harv Rev Psychiatry 1995 2 274 281 10.3109/10673229509017146 9384911

[B38] Mercer D Douglass AB Links PS Meta-analyses of mood stabilizers, antidepressants and antipsychotics in the treatment of borderline personality disorder: effectiveness for depression and anger symptoms J Pers Disord 2009 23 156 174 10.1521/pedi.2009.23.2.156 19379093

[B39] McQuade DV Barrnett RJ King BH Pharmacological intervention in aggression Neurobiology of Aggression: Understanding and Preventing Violence 2003 Totawa, NJ: Humana Press, Inc. 289 313

[B40] Comai S Tau M Pavlovic Z Gobbi G The psychopharmacology of aggressive behavior: a translational approach: part 2: clinical studies using atypical antipsychotics, anticonvulsants, and lithium J Clin Psychopharmacol 2012 32 237 260 10.1097/JCP.0b013e31824929d6 22367663

[B41] Barratt ES Stanford MS Felthous AR Kent TA The effects of phenytoin on impulsive and premeditated aggression: a controlled study J Clin Psychopharmacol 1997 17 341 349 10.1097/00004714-199710000-00002 9315984

[B42] Bowden CL Grunze H Mullen J Brecher M Paulsson B Jones M Vagero M Svensson K A randomized, double-blind, placebo-controlled efficacy and safety study of quetiapine or lithium as monotherapy for mania in bipolar disorder J Clin Psychiatry 2005 66 111 121 10.4088/JCP.v66n0116 15669897

[B43] Yatham LN Paulsson B Mullen J Vagero AM Quetiapine versus placebo in combination with lithium or divalproex for the treatment of bipolar mania J Clin Psychopharmacol 2004 24 599 606 10.1097/01.jcp.0000144887.66319.2f 15538120

[B44] Campbell M Adams PB Small AM Kafantaris V Silva RR Shell J Perry R Overall JE Lithium in hospitalized aggressive children with conduct disorder: a double-blind and placebo-controlled study J Am Acad Child Adolesc Psychiatry 1995 34 445 453 10.1097/00004583-199504000-00011 7751258

[B45] Craft M Ismail IA Krishnamurti D Mathews J Regan A Seth RV North PM Lithium in the treatment of aggression in mentally handicapped patients. A double-blind trial Br J Psychiatry 1987 150 685 689 10.1192/bjp.150.5.685 3115350

[B46] Kafantaris V Coletti DJ Dicker R Padula G Kane JM Lithium treatment of acute mania in adolescents: a large open trial J Am Acad Child Adolesc Psychiatry 2003 42 1038 1045 10.1097/01.CHI.0000070247.24125.24 12960703

[B47] Masi G Milone A Manfredi A Pari C Paziente A Millepiedi S Effectiveness of lithium in children and adolescents with conduct disorder: a retrospective naturalistic study CNS Drugs 2009 23 59 69 10.2165/0023210-200923010-00004 19062775

[B48] Worrall EP Moody JP Naylor GJ Lithium in non-manic-depressives: antiaggressive effect and red blood cell lithium values Br J Psychiatry 1975 126 464 468 10.1192/bjp.126.5.464 1092398

[B49] Jones RM Arlidge J Gillham R Reagu S van den Bree M Taylor PJ Efficacy of mood stabilisers in the treatment of impulsive or repetitive aggression: systematic review and meta-analysis Br J Psychiatry 2011 198 93 98 10.1192/bjp.bp.110.083030 21282779

[B50] Sheard MH Marini JL Bridges CI Wagner E The effect of lithium on impulsive aggressive behavior in man Am J Psychiatry 1976 133 1409 1413 984241 10.1176/ajp.133.12.1409

[B51] Umhau JC Dauphinais KM L'Abate L Omega-3 polyunsaturated fatty acids and health Low-Cost Approaches to Promote Physical and Mental Health: Theory, Research and Practice 2007 New York, NY Springer Publishing 87 101

[B52] Simopoulos AP Evolutionary aspects of diet: the omega-6/omega-3 ratio and the brain Mol Neurobiol 2011 44 203 215 10.1007/s12035-010-8162-0 21279554

[B53] Werbach M Nutritional influences on aggressive behavior J Orthomol Med 1992 7 45 51

[B54] Harris WS Expert opinion: omega-3 fatty acids and bleeding-cause for concern? Am J Cardiol 2007 99 44C 46C 10.1016/j.amjcard.2006.11.021 17368278

[B55] Mozaffarian D Rimm EB Fish intake, contaminants, and human health: evaluating the risks and the benefits JAMA 2006 296 1885 1899 10.1001/jama.296.15.1885 17047219

[B56] Hibbeln JR Ferguson TA Blasbalg TL Omega-3 fatty acid deficiencies in neurodevelopment, aggression and autonomic dysregulation: opportunities for intervention Int Rev Psychiatry 2006 18 107 118 10.1080/09540260600582967 16777665

[B57] Freeman MP Hibbeln JR Wisner KL Davis JM Mischoulon D Peet M Keck PE Jr Marangell LB Richardson AJ Lake J Stoll AL Omega-3 fatty acids: evidence basis for treatment and future research in psychiatry J Clin Psychiatry 2006 67 1954 1967 10.4088/JCP.v67n1217 17194275

[B58] Gesch CB Hammond SM Hampson SE Eves A Crowder MJ Influence of supplementary vitamins, minerals and essential fatty acids on the antisocial behaviour of young adult prisoners randomised, placebo-controlled trial Br J Psychiatry 2002 181 22 28 10.1192/bjp.181.1.22 12091259

[B59] Zaalberg A Nijman H Bulten E Stroosma L van der Staak C Effects of nutritional supplements on aggression, rule-breaking, and psychopathology among young adult prisoners Aggress Behav 2010 36 117 126 10.1002/ab.20335 20014286

[B60] Schoenthaler SJ Bier ID The effect of vitamin-mineral supplementation on juvenile delinquency among American schoolchildren: a randomized, double-blind placebo-controlled trial J Altern Complement Med 2000 6 7 17 10.1089/acm.2000.6.7 10706231

[B61] Murphy CM Ting LA Interventions for perpetrators of intimate partner violence: A review of efficacy research and recent trends Partner Abuse 2010 1 26 44 10.1891/1946-6560.1.1.26

[B62] Clark DB Pharmacotherapy for adolescent alcohol use disorder CNS Drugs 2012 26 559 569 10.2165/11634330-000000000-00000 22676261

[B63] Williams SH Medications for treating alcohol dependence Am Fam Physician 2005 72 1775 1780 16300039

[B64] Ernst DB Pettinati HM Weiss RD Donovan DM Longabaugh R An intervention for treating alcohol dependence: relating elements of Medical Management to patient outcomes with implications for primary care Ann Fam Med 2008 6 435 440 10.1370/afm.884 18779548 PMC2532776

[B65] Babcock JC Green CE Robie C Does batterers' treatment work? A meta-analytic review of domestic violence treatment Clin Psychol Rev 2004 23 1023 1053 10.1016/j.cpr.2002.07.001 14729422

[B66] Chemtob CM Novaco RW Hamada RS Gross DM Cognitive-behavioral treatment for severe anger in posttraumatic stress disorder J Consult Clin Psychol 1997 65 184 189 9103748 10.1037//0022-006x.65.1.184

[B67] Gondolf EW Batterer programs J Interpers Violence 1997 12 83 98 10.1177/088626097012001006

[B68] Lawson DM Dawson TE Kieffer KM Perez LM Burke J Kier FJ An integrated feminist/cognitive-behavioral and psychodynamic group treatment model for women who abuse their partners Psychol Men Masculinity 2001 2 86 99

[B69] Gondolf EW A 30-month follow-up of court-referred batterers in four cities Int J Offender Ther Comp Criminol 2000 44 111 128 10.1177/0306624X00441010

[B70] Beck R Fernandez E Cognitive-behavioral therapy in the treatment of anger: a meta-analysis Cogn Ther Res 1998 22 63 74 10.1023/A:1018763902991

[B71] Coccaro E Aggression: Psychiatric Assessment and Treatment 2003 22 Zug, Switzerland: Informa Healthcare

[B72] Swenson CR Sanderson C Dulit RA Linehan MM The application of dialectical behavior therapy for patients with borderline personality disorder on inpatient units Psychiatr Q 2001 72 307 324 10.1023/A:1010337231127 11525079

[B73] Evershed S Tennant A Boomer D Rees A Barkham M Watson A Practice-based outcomes of dialectical behaviour therapy (DBT) targeting anger and violence, with male forensic patients: a pragmatic and non-contemporaneous comparison Crim Behav Ment Health 2003 13 198 213 10.1002/cbm.542 14654871

[B74] Maiuro RD Eberle JA State standards for domestic violence perpetrator treatment: current status, trends, and recommendations Violence Vict 2008 23 133 155 10.1891/0886-6708.23.2.133 18624086

[B75] Levesque DA Ciavatta MM Castle PH Prochaska JM Prochaska JO Evaluation of a stage-based, computer-tailored adjunct to usual care for domestic violence offenders Psychol Violence 2012 1 10.1037/a0027501 PMC3569030 23412627

[B76] Taft CT Murphy CM Elliott JD Morrel TM Attendance-enhancing procedures in group counseling for domestic abusers J Couns Psychol 2001 48 51 60

[B77] Horvath AO Symonds BD Relation between working alliance and outcome in psychotherapy: a meta-analysis J Couns Psychol 1991 38 139149

[B78] Stone AA The Tarasoff decisions: suing psychotherapists to safeguard society Harv Law Rev 1976 358 378 1028678

[B79] Walcott DM Cerundolo P Beck JC Current analysis of the Tarasoff duty: an evolution towards the limitation of the duty to protect Behav Sci Law 2001 19 325 43 10.1002/bsl.444 11443695

[B80] Kaur G Herbert L Recognizing and intervening in intimate partner violence Cleve Clin J Med 2005 72 406 409 10.3949/ccjm.72.5.406 15929454

[B81] Brown JL Physicians have ethical, legal obligation to report child abuse AAP News 2012 33 20 20

[B82] Houry D Sachs CJ Feldhaus KM Linden J Violence-inflicted injuries: reporting laws in the fifty states Ann Emerg Med 2002 39 56 60 10.1067/mem.2002.117759 11782731

[B83] Geiderman J Jesus J, Rosen P, Adams J, Derse AR, Grossman SA, Wolfe R Mandatory and permissive reporting laws: conflicts in patient confidentiality, autonomy, and the duty to report Ethical Problems in Emergency Medicine: a Discussion-based Review 2012 1 Hoboken, NJ: Wiley-Blackwell 271 285

[B84] Felthous AR The clinician's duty to protect third parties Psychiatr Clin North Am 1999 22 49 60 10.1016/S0193-953X(05)70058-7 10083944

[B85] Beck JC Legal and ethical duties of the clinician treating a patient who is liable to be impulsively violent Behav Sci Law 1998 16 375 89 10.1002/(SICI)1099-0798(199822)16:3<375::AID-BSL312>3.0.CO;2-J 9768467

[B86] Yudofsky S Stevens L Silver J Barsa J Williams D Propranolol in the treatment of rage and violent behavior associated with Korsakoff's psychosis Am J Psychiatry 1984 141 114 115 6691426 10.1176/ajp.141.1.114

[B87] Látalová K Bipolar disorder and aggression Int J Clin Pract 2009 63 889 899 10.1111/j.1742-1241.2009.02001.x 19490199

[B88] Boles SM Miotto K Substance abuse and violence: a review of the literature Aggress Violent Behav 2003 8 155 174 10.1016/S1359-1789(01)00057-X

[B89] Tuninger EE Levander S Bernce R Johansson G Criminality and aggression among psychotic in-patients: frequency and clinical correlates Acta Psychiatr Scand 2001 103 294 300 10.1034/j.1600-0447.2001.00028.x 11328244

[B90] d'Orbán PT Dalton J Violent crime and the menstrual cycle Psychol Med 1980 10 353 359 10.1017/S0033291700044123 7189893

[B91] Barlow DH Anxiety and its Disorders The Nature and Treatment of Anxiety and Panic 2004 New York, NY: The Guilford Press

[B92] Kernberg OF Aggression in Personality Disorders and Perversions 1995 New Haven, CT: Yale University Press

[B93] Loney J Lahey BB, Kazdin AE Hyperactivity and aggression in the diagnosis of attention deficit disorder Advances in clinical child psychology 1987 10 New York, NY: Plenum Press 99 135

[B94] Feldbau-Kohn S Heyman RE O'Leary KD Major depressive disorder and depressive symptomatology as predictors of husband to wife physical aggression Violence Vict 1998 13 347 360 10328443

[B95] Villano JL Mlinarevich N Watson KS Engelhard HH Anderson-Shaw L Aggression in a patient with primary brain tumor: ethical implications for best management J Neuro-Oncol 2009 94 293 296 10.1007/s11060-009-9850-3 19267227

[B96] Dickson DW Davies P Mayeux R Crystal H Horoupian DS Thompson A Goldman JE Diffuse Lewy body disease Acta neuropathologica 1987 75 8 15 10.1007/BF00686786 3434218

[B97] Benedict RH Shapiro A Priore R Miller C Munschauer F Jacobs L Neuropsychological counseling improves social behavior in cognitively-impaired multiple sclerosis patients Mult Scler 2000 6 391 396 11212135 10.1177/135245850000600606

[B98] Kito Y Kazui H Kubo Y Yoshida T Takaya M Wada T Nomura K Hashimoto M Ohkawa S Miyake H Neuropsychiatric symptoms in patients with idiopathic normal pressure hydrocephalus Behav Neurol 2009 21 165 174 19996513 10.3233/BEN-2009-0233 PMC5444276

[B99] Rosenblatt A Leroi I Neuropsychiatry of Huntington's disease and other basal ganglia disorders Psychosomatics 2000 41 24 30 10.1016/S0033-3182(00)71170-4 10665265

[B100] Orengo CA Khan J Kunik ME Snow AL Morgan R Steele A Cully JA Graham DP Aggression in individuals newly diagnosed with dementia Am J Alzheimers Dis Other Demen 2008 23 227 232 10.1177/1533317507313373 18258723 PMC10846265

[B101] Golden CJ Jackson ML Peterson-Rohne A Gontkovsky ST Neuropsychological correlates of violence and aggression: A review of the clinical literature Aggress Violent Beh 1996 1 3 25 10.1016/1359-1789(95)00002-X

[B102] Rayel MG Land WB Gutheil TG Dementia as a risk factor for homicide J Forensic Sci 1999 44 565 567 10408111

[B103] Hector M Burton JR What are the psychiatric manifestations of vitamin B_12 _deficiency? J Am Geriatr Soc 1988 36 1105 1112 3057051 10.1111/j.1532-5415.1988.tb04397.x

[B104] Lonsdale D Shamberger RJ Red cell transketolase as an indicator of nutritional deficiency Am J Clin Nutr 1980 33 205 211 7355794 10.1093/ajcn/33.2.205

[B105] Werbach M Nutritional influences on aggressive behavior J Orthomol Med 1992 7 45 51

[B106] Liu J Raine A The effect of childhood malnutrition on externalizing behavior Curr Opin Pediatr 2006 18 565 570 10.1097/01.mop.0000245360.13949.91 16969174

[B107] Schrauzer GN Shrestha KP Lithium in drinking water and the incidences of crimes, suicides, and arrests related to drug addictions Biol Trace Elem Res 1990 25 105 113 10.1007/BF02990271 1699579

[B108] Rasmussen HH Mortensen PB Jensen IW Depression and magnesium deficiency Int J Psychiatry Med 1989 19 57 63 10.2190/NKCD-1RB1-QMA9-G1VN 2722406

[B109] Branchey L Branchey M Shaw S Lieber CS Depression, suicide, and aggression in alcoholics and their relationship to plasma amino acids Psychiatry Res 1984 12 219 226 10.1016/0165-1781(84)90027-1 6593754

[B110] Giancola PR Corman MD Alcohol and aggression: a test of the attention-allocation model Psychol Sci 2007 18 649 655 10.1111/j.1467-9280.2007.01953.x 17614875

[B111] Licata A Taylor S Berman M Cranston J Effects of cocaine on human aggression Pharmacol Biochem Behav 1993 45 549 552 10.1016/0091-3057(93)90504-M 8332615

[B112] Gottschalk LA Rebello T Buchsbaum MS Tucker HG Hodges EL Abnormalities in hair trace elements as indicators of aberrant behavior Compr Psychiatry 1991 32 229 237 10.1016/0010-440X(91)90043-C 1884602

[B113] Singh S Sharma N Neurological syndromes following organophosphate poisoning Neurol India 2000 48 308 313 11146591

[B114] Henry J Hopkins PM, Ellis FR Drug overdose, drugs of abuse and hypermetabolism Hyperthermic and Hypermetabolic Disorders: Exertional Heat-stroke, Malignant Hyperthermia and Related Syndromes 1996 Cambridge, UK: Press Syndicate of the University of Cambridge 239 258

[B115] Blacker K Aggression and the chronic use of LSD J Psychoactive Drugs 1970 3 32 37 10.1080/02791072.1970.10471359

[B116] Gerra G Zaimovic A Raggi MA Giusti F Delsignore R Bertacca S Brambilla F Aggressive responding of male heroin addicts under methadone treatment: psychometric and neuroendocrine correlates Drug Alcohol Depend 2001 65 85 95 10.1016/S0376-8716(01)00152-1 11714593

[B117] Anderson PD Bokor G Forensic Aspects of Drug-Induced Violence Journal of Pharmacy Practice 2012 25 41 49 10.1177/0897190011431150 22215647

[B118] DiMascio A The effects of benzodiazepines on aggression: reduced or increased? Psychopharmacology 1973 30 95 102 10.1007/BF00421423 4576033

[B119] Williams ER Shepherd SM Medical clearance of psychiatric patients Emerg Med Clin North Am 2000 18 185 198 10.1016/S0733-8627(05)70117-6 10767877

[B120] Benton D Kumari N Brain PF Mild hypoglycaemia and questionnaire measures of aggression Biol Psychol 1982 14 129 135 10.1016/0301-0511(82)90020-5 7104424

[B121] Rahman T Hodgson H Clinical management of acute hepatic failure Intensive Care Med 2001 27 467 476 10.1007/s001340100873 11355114

[B122] Gupta N Midha P Singh G Gupte S Psychiatric aspects of endocrine disorders Recent Advances Pediatrics: Pediatric Endocrinology. Special Volume 13 2004 New Delhi: Jaypee Brothers Medical Publishers 308

[B123] Trafford PA Homicide in acute porphyria Forensic Sci 1976 7 113 120 10.1016/0300-9432(76)90027-3 964803

[B124] Berkowitz L Pain and aggression: some findings and implications Motivation Emotion 1993 17 277 293 10.1007/BF00992223

[B125] Soldatos C Kales A Sleep disorders: research in psychopathology and its practical implications Acta Psychiatr Scand 1982 65 381 387 10.1111/j.1600-0447.1982.tb00862.x 7124421

[B126] Arai A Ishii T Yasui T Takahashi A Ishigooka J Murakaki M A case of iron deficiency anemia with delirium Kanagawaken Seishin Igakkaishi 1998 48 31 36

[B127] Simpson TL Saxon AJ Meredith CW Malte CA McBride B Ferguson LC Gross CA Hart KL Raskind M A pilot trial of the alpha-1 adrenergic antagonist, prazosin, for alcohol dependence Alcohol Clin Exp Res 2009 33 255 263 10.1111/j.1530-0277.2008.00807.x 18945226

[B128] Kaplan BJ Crawford SG Gardner B Farrelly G Treatment of mood lability and explosive rage with minerals and vitamins: two case studies in children J Child Adolesc Psychopharmacol 2002 12 205 219 10.1089/104454602760386897 12427294

[B129] Yudofsky S Williams D Gorman J Propranolol in the treatment of rage and violent behavior in patients with chronic brain syndromes Am J Psychiatry 1981 138 218 220 7457643 10.1176/ajp.138.2.218

[B130] Stanford MS Houston RJ Mathias CW Greve KW Villemarette-Pittman NR Adams D A double-blind placebo-controlled crossover study of phenytoin in individuals with impulsive aggression Psychiatry Res 2001 103 193 203 10.1016/S0165-1781(01)00287-6 11549407

[B131] Coccaro EF Kavoussi RJ Fluoxetine and impulsive aggressive behavior in personality-disordered subjects Arch Gen Psychiatry 1997 54 10811088 10.1001/archpsyc.1997.01830240035005 9400343

[B132] George DT Phillips MJ Lifshitz M Lionetti TA Spero DE Ghassemzedeh N Doty L Umhau JC Rawlings RR Fluoxetine treatment of alcoholic perpetrators of domestic violence: a 12-week, double-blind, randomized, placebo-controlled intervention study J Clin Psychiatry 2011 72 60 65 10.4088/JCP.09m05256gry 20673556 PMC3026856

[B133] Sultzer DL Gray KF Gunay I Berisford MA Mahler ME A double-blind comparison of trazodone and haloperidol for treatment of agitation in patients with dementia Am J Geriatr Psychiatry 1997 5 60 69 9169246

[B134] Buckley PF The role of typical and atypical antipsychotic medications in the management of agitation and aggression J Clin Psychiatry 1999 60 suppl 10 52 60 10340688

